# LFP-Based Gravure Printed Cathodes for Lithium-Ion Printed Batteries

**DOI:** 10.3390/membranes9060071

**Published:** 2019-06-07

**Authors:** Maria Montanino, Giuliano Sico, Anna De Girolamo Del Mauro, Margherita Moreno

**Affiliations:** 1 ENEA Italian National Agency for New Technologies, Energy and Sustainable Economic Development, SSPT-PROMAS-NANO, 80055 Portici, Italy; giuliano.sico@enea.it (G.S.); anna.degirolamo@enea.it (A.D.G.D.M.); 2 ENEA Italian National Agency for New Technologies, Energy and Sustainable Economic Development, DTE-PCU-SPCT, 00123 Roma, Italy; margherita.moreno@enea.it

**Keywords:** gravure printing, printed batteries, printed cathode, lithium batteries, multilayer

## Abstract

Printed batteries have undergone increased investigation in recent years because of the growing daily use of small electronic devices. With this in mind, industrial gravure printing has emerged as a suitable production technology due to its high speed and quality, and its capability to produce any shape of image. The technique is one of the most appealing for the production of functional layers for many different purposes, but it has not been highly investigated. In this study, we propose a LiFePO_4_ (LFP)-based gravure printed cathode for lithium-ion rechargeable printed batteries and investigate the possibility of employing this printing technique in battery manufacture.

## 1. Introduction

The use of printing techniques as a low cost production method for creating layers of different functional materials has recently undergone increased investigation in many fields. Compared with coating techniques, printing allows greater control of the characteristics of the layer, the possibility to realize any desired shape and pattern, and it also delivers a higher production speed. Among these printing techniques, the gravure technique is the most commonly used for the production of magazines and flexible packaging because of its ability to couple high throughput (speed up to 400 m/min^−1^) and high quality (resolution 0.1 µm). Gravure is considered to be the most promising technique for producing thin layers (0.05–10 µm) of different functional materials [1]. The use of such conventional roll-to-roll industrial printing techniques allows the manufacture of low cost flexible structures and devices at high volume [2,3] in a one-step direct deposition process, which is suitable for patterning realization and large area production under ambient conditions, coupled with a minimal waste of energy, time, and materials [4,5,6]. With this aim, in the last few years organic materials, such as polymers and conductive polymers, have been successfully gravure printed in our laboratories to be employed in the field of optoelectronic [7,8,9,10]. More recently, this technique has also been demonstrated to be suitable for inorganic materials such as ceramics, offering the possibility to tailor the layer’s characteristics through the modulation of the printing parameters [11]. The level of control of particle deposition is high enough to allow an innovative method of oxide sintering at low temperatures under pressure-less conditions [12].

In this paper, we demonstrate that it is possible to employ the gravure printing technique in the field of printed batteries. Printed batteries are thin batteries used in portable electronic devices, and their use is becoming more and more widespread in our daily lives [13]. All such devices (e.g., wearable, beauty, and biomedical) need only a small specific capacity (5–10 mAh·cm^−2^), which has to be provided by a thin and customizable battery with a volume below 10 mm^3^ in order for it to be perfectly integrated into the device. To date, industrially produced printed batteries are mostly not rechargeable [13].

Despite its possible advantages, the use of gravure printing for the production of printed batteries has not been well reported in scientific literature [14]. This is mainly due to the requirement of having to use low viscosity inks in order to achieve adequate thickness, particularly in electrodes, for achieving adequate capacities [15]. In addition, the possible contamination of materials from the printing cylinder (steel, copper, or chromium) limits the ink formulation. Finally, while the possibility to print polymers and inorganic materials separately has been demonstrated, the possibility to print such materials together in a homogenous composite structure remains a challenge.

In this study, gravure printing is used to produce LiFePO_4_ (LFP)-based cathodes for rechargeable lithium-ion batteries using a multilayer approach. The well-known LFP was chosen as a reference. Moreover, in accordance with the most recent research regarding green aspects of component preparation, the cathodes were prepared using a water soluble sodium carboxy methyl cellulose (CMC) binder, which was deposited by water based ink solution.

## 2. Materials and Methods

Suitable inks were prepared for the gravure printing of the cathodes, with a fixed percentage of the solid component and a variable solvent content. The materials involved and their proportions were as follows: LiFePO_4_ (LFP) (Sigma–Aldrich, Milan, Italy) as the active material (84%), super P (Thermofisher, Karlsruhe, Germany) as the conductive carbon (10%), and sodium carboxy methyl cellulose (CMC) (Panreac Quimica sa., Barcelona, Spain) as the binder (6%). The solvent used was a mixture of water and 2-propanol (80–20 wt%). The cathodes were constructed through a multilayer deposition of 3 layers (3L) or 5 layers (5L). The first layer was deposited by ink containing 23% by weight of dry content. The second layer was deposited by adding 10% of the mixed solvent to the ink used for the first layer. The successive layers were printed, adding a further 5% of the mixed solvent in each step. The solid content of each layer is reported in Table 1. The cathodic layers were deposited on aluminum foils (Sigma–Aldrich) using a commercial lab-scale IGT G1-5 gravure printer (IGT, Alemere, Netherlands) equipped with a cylinder with a line density of 40 lines/cm, stylus angle of 120°, cell depth of 72 µm, and screen angle of 53°. Each layer, and the finished cathodes, were dried at 130 °C. No final calendering was performed on the printed cathodes. After preliminary tests, the best printing conditions were found to be a printing force of 500 N at a speed of 36 m/min. The printing conditions were kept constant for all the printed layers. The electrical conductivity of the printed layers was verified by sheet resistance measurements performed by a four points probe instrument (Resistest RT 8A coupled with Resistage RG 8 supplied by Napson, Korea). The thickness and surface roughness of the printed samples were investigated by interferometry-based optical profilometer (Talysurf CCI HD, Taylor Hobson, Leicester, UK). The reported values represent the average obtained by several measurements and have a standard deviation of about 10%. The root mean square surface roughness was obtained according to the ISO 25178 standard. The morphology of the printed cathodes was also investigated through scanning electron microscopy (1530, LEO Elektronenmikroskopie GmbH, Oberkochen, Germany). The 3L and 5L cathodes were cut into discs of 14 mm diameter and tested in cells against lithium metal foil discs of 12 mm diameter. The separator was a glass fiber disc and the electrolyte used was an LP30 battery grade (Sigma–Aldrich) (1 M solution LiPF_6_ in a 1:1 by volume mixture of ethylene carbonate and diethyl carbonate (EC:DEC, 1:1)). Galvanostatic cycling measurements were performed on the cells by a Maccor 4000 at 20 °C, at a fixed 0.1 C, and then at increasing C-rates.

## 3. Results and Discussion

**Figure 1:**
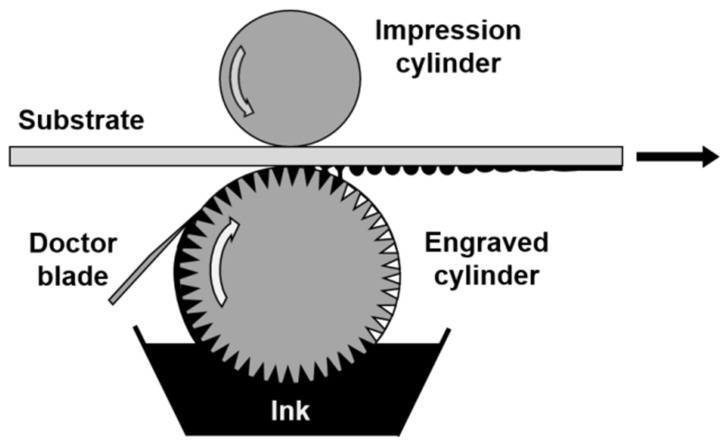
Schematic of the gravure printing process.

The gravure printing process consists of the fluid transfer of low-viscosity ink from the micro-engraved cells of a printing cylinder directly onto a flexible substrate through the pressure of a rubber cylinder as depicted in Figure 1. The desired geometry/patterning is obtained by engraving it onto the printing cylinder. 

**Figure 2:**
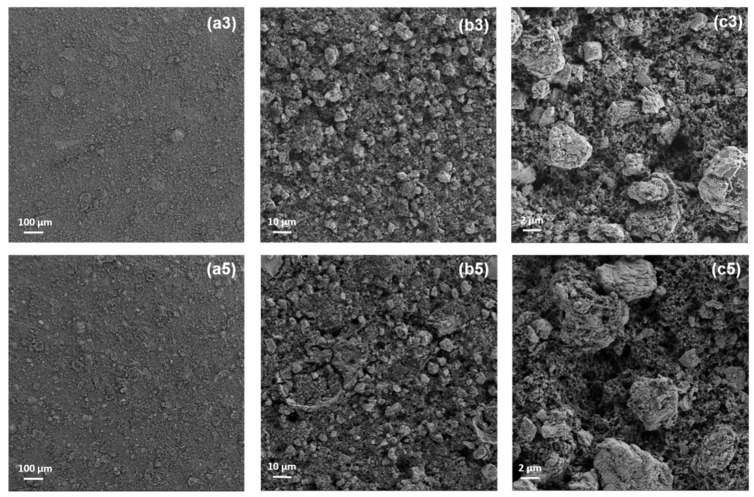
SEM images of the 3 layer (3L) (**a3**,**b3**,**c3**) and 5 layer (5L) (**a5**,**b5**,**c5**) gravure printed cathodes at different magnifications.

Several physical parameters relating to the materials, such as the ink and the substrate, are important in the gravure printing production quality; the ink viscosity, its rheological behavior, the surface tension/surface energy, the solvent evaporation rate, and the substrate porosity and smoothness. Moreover, process parameters such as cell geometry and density, printing pressure, and speed also play an important role on the final results. Although it may appear to be a relatively simple process, gravure printing has a complex multi-physical nature involving a series of sub-processes (inking, doctoring, transfer, spreading, and drying), each with its ideal operating regime, and each one determining the final quality of the printed product. In addition, an important issue is the formulation of low viscosity inks (1–100 mPa·s) [16] suitable for gravure printing that are able to realize proper functional layers. Thus, in order to prepare the inks, a large quantity of solvent is required. In this work, in order to develop a sustainable process, a water soluble CMC was used as a binder, and for this reason a mixture of water and 2-propanol was used in the ink formulation. The 2-propanol played the role of improving the ink printability, decreasing the surface tension due to the use of water, and improving the ink wettability of both the substrate and the printing cylinder. Taking into account all such matters, several preliminary tests were carried out to identify the best ink composition and process parameters, and the results are reported in the experimental section. Composite cathodic materials were successfully gravure printed onto aluminum foils and demonstrated good printability. To increase the mass loading, a multilayer approach was used. Up to five layers were overlapped using inks at decreasing dry content levels, keeping all the other printing parameters (cylinder, speed, pressure, drying temperature) constant, which benefited the overall production process. The multilayer was created by stacking at increasing solvent amounts in order to improve the distribution. This approach has been proved as the best way to lay down the consecutive printed layers [8]. The layer by layer characteristics of the printed cathodes are reported in Table 1. The mass loading of the active LFP material increased until the third layer. When adding another two layers (up to five), the increase in the mass loading and the thickness of the printed layers was poor due to the decreasing dry content of the inks. It had been expected that the increase in the solvent content of the inks on each of the layers would restrain the surface roughness increase, but this effect was not observed. This is likely due to the starting size distribution of the active material, which was measured in microns. In addition, the SEM images in Figure 2 show worse distribution and a slightly higher inhomogeneity in the 5L cathode when compared with the 3L.

**Figure 3:**
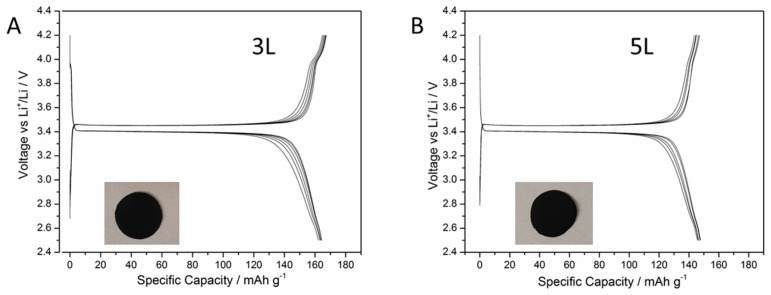
The galvanostatic profiles for cycles 5–10 obtained for the 3L (**A**) and the 5L (**B**) gravure printed cathode. The insets are photographs of the electrodes.

When the magnification of the cathode images is increased, it can be seen that the lower homogeneity of the 5L appears to be caused by polymer segregation occurring between the CMC polymer and the LFP active material. This is probably due to the low affinity of the CMC versus the increasing 2-propanol content in the ink generating the formation of polymer domains into the printed layer, thus worsening the distribution of the components in the 5L cathode itself. Both the cathodes were tested in batteries against lithium metal. In Figure 3, examples of charge-discharge cycles obtained for the 3L and 5L cathodes are reported.

**Figure 4:**
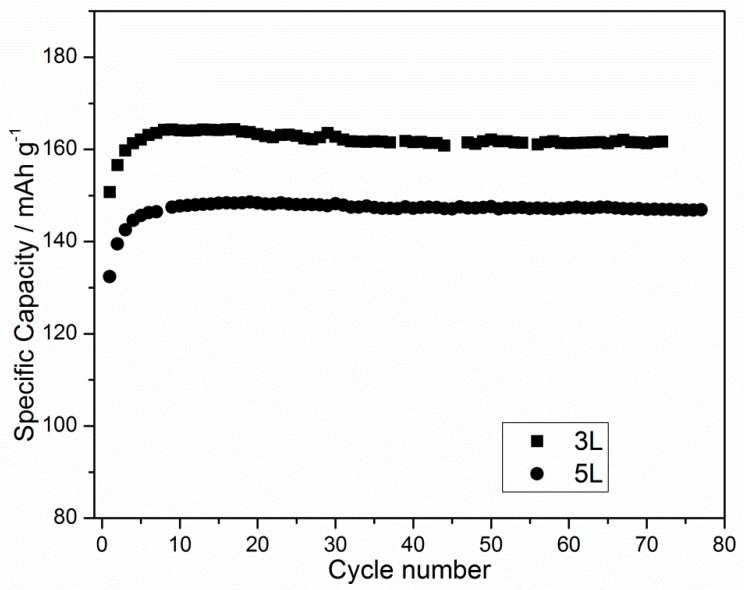
The discharge specific capacity vs. cycle number of the 3L and 5L gravure printed cathodes.

The galvanostatic profiles appear featureless and present a typical LFP plateau, which is flat around 3.4 V in both charge and discharge, showing a stable cyclability and specific capacities close to the theoretical one (170 mAh/g). This is especially true for the 3L cathode. These results demonstrate that the structure of the printed layer is suitable to be used as a cathode. The discharge specific capacities of the 3L and 5L cathodes are shown in Figure 4. 

Sample 3L shows specific capacity values close to the theoretical ones, with a very high coulombic efficiency (>98%) for almost 100 cycles. After a few initialization cycles (<5) there is no capacity fading upon cycling at C/10. The same behavior can be observed in the 5L sample, but the values are 20 mAh lower. This provides important feedback regarding the production process. The higher homogeneity of the 3L cathode leads to higher efficiency in its behavior in batteries, thus demonstrating that the 3L cathode is better than the 5L cathode, especially when considering the necessary production steps. Since no calendering was performed on the printed cathodes, the positive battery tests suggest that using gravure printing would allow such post-process steps to be skipped, which would simplify the overall process. Cycling of the investigated cells was continued, and they showed good stability over time. Therefore, long life cyclability can be expected.

**Figure 5:**
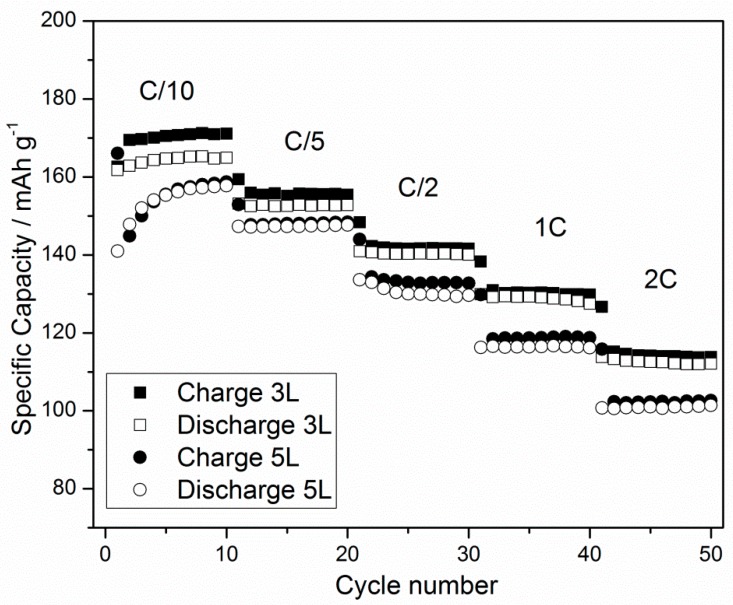
The charge-discharge specific capacity vs. the cycle number of the 3L and 5L gravure printed cathodes at increasing C-rates.

The charge and discharge capacities compared to the cycle numbers at increasing specific currents for the cells containing the 3L and 5L cathodes are reported in Figure 5. The Figure shows good stability of the cells at different rates and similar values for the charge and discharge capacities above 100 mAh·g^−1^, even at 2 C-rate. However, the obtained mass loadings (see Table 1) are too low for practical applications, but they could be improved by increasing the thickness and density of the layers by decreasing the size and narrowing the size distribution of the active material. This would allow the printability of more concentrated inks, even when using different multilayer profiles. Moreover, a substrate pre-treatment, such as a commonly used corona discharge, would also improve the distribution of the solid on the substrate. Such changes would also improve the homogeneity of the printed electrode, further improving its performances. Furthermore, the use of a better performing active material than LFP would simplify the target achievement. Nevertheless, the good performance results, in terms of efficiency and reproducibility, of the printed cathodes in this study prove the feasibility of gravure printing in the field of printed batteries. This work may open the way for layer by layer device manufacturing using only gravure printing, which would potentially bring large advantages in terms of fast, easy, and low cost printed battery production.

## 4. Conclusions

Thanks to a multilayer approach, the gravure printing technique led to the production of functional composite layers. The feasibility to gravure print cathodes for batteries has been demonstrated and, even with only a few overlapped layers, good performances was achieved. Keeping most of the printing parameters constant during the production process and skipping the calendering step allowed the manufacturing process to be simplified, which would make its industrial scaling easier. The performances of the printed cathodes could be improved by increasing the layer homogeneity by decreasing the size and the size distribution of the starting materials. These results open the way to the possibility of utilizing such techniques in future industrial production, especially in the field of printed batteries.

## Figures and Tables

**Table 1:** Layer by layer characteristics of the gravure printed cathodes.

Layer n.	Ink dry Content (%)	Overall Active Material (g cm^−2^)	Overall Thickness (µm)	Surface Roughness (nm)
1	23	9 10^−5^	1.7	1
2	21	2 10^−4^	3.2	1.5
3	20	4 10^−4^	4.6	1.8
4	19	5 10^−4^	5.9	2.3
5	18	5 10^−4^	7.1	2.5

## References

[1] Søndergaard R.R., Hosel M., Krebs F.C. (2013). Roll-to-Roll Fabrication of Large Area Functional Organic Materials. J. Polym. Sci. Part B Polym. Phys..

[2] Tsay C.-Y., Wu P.-W. (2013). Low temperature deposition of ZnO semiconductor thin films on a PEN substrate by a solution process. Electron. Mater. Lett..

[3] Kim S.J., Yoon S., Kim H.J. (2014). Review of solution-processed oxide thin-film transistors. Jpn. J. Appl. Phys..

[4] Puetz J., Aegerter M.A. (2008). Direct gravure printing of indium tin oxide nanoparticle patterns on polymer foils. Thin Solid Films.

[5] Alsaid D.A., Rebrosova E., Joyce M., Rebros M., Atashbar M., Bazuin B. (2012). Gravure printing of ITO transparent electrodes for applications in flexible electronics. J. Display Technol..

[6] Khan S., Lorenzelli L., Dahiya R. (2015). Technologies for printing sensors and electronics over large flexible substrates: A review. IEEE Sens. J..

[7] Montanino M., De Girolamo Del Mauro A., Tesoro M., Ricciardi R., Diana R., Morvillo P., Nobile G., Imparato A., Sico G., Minarini C. (2015). Gravure-printed PEDOT:PSS on flexible PEN substrate as ITO-free anode for polymer solar cells. Polym. Compos..

[8] Sico G., Montanino M., De Girolamo Del Mauro A., Imparato A., Nobile G., Minarini C. (2016). Effects of the ink concentration on multi-layer gravure-printed PEDOT:PSS. Org. Electron..

[9] Montanino M., Sico G., Prontera C.T., De Girolamo Del Mauro A., Aprano S., Maglione M.G., Minarini C. (2017). Gravure printed PEDOT:PSS as anode for flexible ITO-free organic light emitting diodes. Express Polym. Lett..

[10] Sico G., Montanino M., De Girolamo Del Mauro A., Minarini C. (2018). Improving the gravure printed PEDOT:PSS electrode by gravure printing DMSO post-treatment. J. Mater. Sci. Mater. Electron..

[11] Sico G., Montanino M., Prontera C.T., De Girolamo Del Mauro A., Minarini C. (2018). Gravure printing for thin film ceramics manufacturing from nanoparticles. Ceram. Int..

[12] Sico G., Montanino M., Ventre M., Mollo V., Prontera C.T., Minarini C., Magnani G. (2019). Pressureless sintering of ZnO thin film on plastic substrate via vapor annealing process at near-room temperature. Scr. Mater..

[13] Oliveira J., Costa C.M., Lanceros-Méndez S., Lanceros-Méndez S., Costa C.M. (2018). Printed Batteries: An Overview. Printed Batteries Materials, Technologies and Applications.

[14] Hwang S.S., Cho C.G., Park K.-S. (2011). Stabilizing LiCoO_2_ electrode with an overlayer of LiNi_0.5_Mn_1.5_O_4_ by using a Gravure printing method. Electrochem. Commun..

[15] Rassek P., Wendler M., Krebs M., Lanceros-Méndez S., Costa C.M. (2018). Industrial Perspective on Printed Batteries. Printed Batteries Materials, Technologies and Applications.

[16] Krebs F.C. (2009). Fabrication and processing of polymer solar cells: A review of printing and coating techniques. Sol. Energy Mater. Sol. Cells.

